# Intelligent Method for Diagnosing Structural Faults of Rotating Machinery Using Ant Colony Optimization

**DOI:** 10.3390/s110404009

**Published:** 2011-04-06

**Authors:** Ke Li, Peng Chen

**Affiliations:** Department of Environmental Science and Engineering, Faculty of Bioresources, Mie University, 1577 Kurimamachiya-cho, Tsu-shi, Mie-ken, 514-8507, Japan; E-Mail: dayanlv@live.cn

**Keywords:** rotating machinery, structural fault, relative ratio symptom parameter, ant colony optimization

## Abstract

Structural faults, such as unbalance, misalignment and looseness, *etc.*, often occur in the shafts of rotating machinery. These faults may cause serious machine accidents and lead to great production losses. This paper proposes an intelligent method for diagnosing structural faults of rotating machinery using ant colony optimization (ACO) and relative ratio symptom parameters (RRSPs) in order to detect faults and distinguish fault types at an early stage. New symptom parameters called “relative ratio symptom parameters” are defined for reflecting the features of vibration signals measured in each state. Synthetic detection index (*SDI*) using statistical theory has also been defined to evaluate the applicability of the RRSPs. The *SDI* can be used to indicate the fitness of a RRSP for ACO. Lastly, this paper also compares the proposed method with the conventional neural networks (NN) method. Practical examples of fault diagnosis for a centrifugal fan are provided to verify the effectiveness of the proposed method. The verification results show that the structural faults often occurring in the centrifugal fan, such as unbalance, misalignment and looseness states are effectively identified by the proposed method, while these faults are difficult to detect using conventional neural networks.

## 1. Introduction

When building an intelligent system for condition diagnosis of plant machinery, symptom parameters (SPs) are required to express the information indicated by a signal measured for diagnosing machine faults. A good symptom parameter can correctly reflect states and condition trends of plant machinery [1–5]. However, if the rotation speed and load of the plant machinery vary while vibration signals are being measured and a fault is in an early stage, the signal contains strong noise. If the power of the noise is stronger than that of the actual failure signal, misrecognition of useful information for the condition diagnosis may result, and the relationships between symptoms and failure types become ambiguous.

Although many studies on intelligent condition diagnosis for plant machinery have been carried out using techniques such as neural networks (NN), support vector machines (SVM), *etc.* [6–12], these methods cannot solve ambiguous diagnosis problems. In many cases, the neural networks or support vector machines never converge when the learning set data have ambiguity [13].

Ant colony optimization (ACO) is a new simulative evolutionary algorithm that is also called ant colony system (ACS) [14]. ACO was first used for solving the traveling salesman problem (TSP) [15], and it has been successfully applied to a large number of difficult optimization problems, like the quadratic assignment problem (QAP) [16], routing in telecommunication networks, graph coloring problems, scheduling, *etc.* In recent years, ACO also has been applied to the clustering analysis problem and has achieved excellent results. ACO is a kind of simulated evolutionary algorithm based on the positive feedback principle of information. It is strong in terms of robustness and can collect and classify all data according to the amount of information around the clustering center [17–20]. If the state identification for the condition diagnosis of plant machinery can be converted to a clustering problem of the feature patterns of vibration signals measured in different states of rotating machinery, the condition diagnosis by ACO is possible.

The faults (such as unbalance, misalignment or looseness, *etc.*) occurring in a rotating machine with a feature spectrum in the low frequency area are called “structural faults”. Structural faults drag shafts into excessive fatigue, and are the main reason of subsequent failures, such as bearing and gear ones, *etc.* That is to say, structural faults can cause the machinery system to break down and may lead to serious human and economic losses. Therefore, detecting and distinguishing structural faults are extremely important for guaranteeing production efficiency and plant safety.

**Figure 1.**
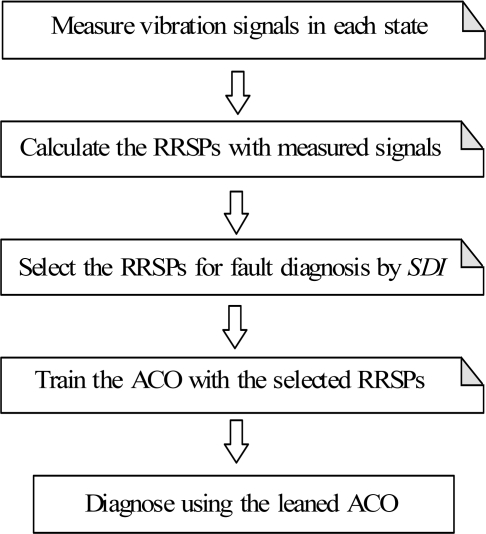
Flowchart of the condition diagnosis.

For the above reasons, this paper proposes a novel method of intelligent condition diagnosis for rotating machinery developed by using relative ratio symptom parameters (RRSPs) and ant colony optimization (ACO). The RRSPs in the low-frequency domain are defined to reflect the features of vibration signals measured in each state. A synthetic detection index (*SDI*) using statistical theory has also defined to evaluate the applicability of the RRSPs for the condition diagnosis. The *SDI* can be used to indicate the fitness of a RRSP for ACO. Moreover, to reduce the convergence time of ACO to increase the processing efficiency, the method of local search for the ACO is also presented in this paper. A practical example of condition diagnosis for a centrifugal fan verifies that the method is effective, and the proposed method is compared with conventional a NN. The flowchart of the condition diagnostic procedure proposed in this paper is shown in Figure 1.

## 2. Relative Ratio Symptom Parameters (RRSPs) for Fault Diagnosis

Many symptom parameters have been defined in the pattern recognition field, in this paper through analyzing the spectral features of structural faults of rotating machinery, the nine RRSPs in the low-frequency domain for structural faults diagnosis of rotating machinery are defined:

(1) P1=Pd(fr)∑i=220Pd(i⋅fr)Pn(fr)∑i=220Pn(i⋅fr)

(2) $$P_{2}=\frac{P_{d}\;\left(2f_{r}\right)/P_{d}\;\left(f_{r}\right)}{P_{n}\;\left(2f_{r}\right)/P_{n}\;\left(f_{r}\right)}$$

(3) $$P_{3}=\frac{P_{d}\;\left(3f_{r}\right)/P_{d}\;\left(f_{r}\right)}{P_{n}\;\left(3f_{r}\right)/P_{n}\;\left(f_{r}\right)}$$

(4) P4=∑i=410Pd(i⋅fr)Pd(fr)∑i=410Pn(i⋅fr)Pn(fr)

where, *f_r_* is the rotating frequency. *P_n_(f_r_)* and *P_d_(f_r_)* are the spectrum values at frequency *f_r_* in the normal state and abnormal states, respectively; *P_n_(if_r_)* and *P_d_(if_r_)* are the high-order harmonic spectrum values at frequency *if_r_* (*i* = 1 to 10) in the normal state and abnormal states, respectively.

(5) P5=∑fi≠i⋅frfi>6frPd(fi)∑i=16Pd(i⋅fr)∑fi≠i⋅frfi>6frPn(fi)∑i=16Pn(i⋅fr)

(6) P6=∑fi>0.6kHfi<1.5kHPd(fi)∑fi≤0.6kHPd(fi)∑fi>0.6kHfi<1.5kHPn(fi)∑fi≤0.6kHPn(fi)

(7) $$P_{7}=\frac{A_{\mathrm{sd}}/A_{\mathrm{hd}}}{A_{\mathrm{sn}}/A_{\mathrm{hn}}}$$

Here, *f_i_* is the frequency and from 0 Hz to the maximum analysis frequency; *A_sn_* and *A_hn_* are the root mean square values of vibration signals of the shaft direction and the horizontal direction in the normal state, respectively; *A_sd_* and *A_hd_* are the root mean square values of vibration signals of the shaft direction and the horizontal direction in the abnormal states, respectively.

(8) $$P_{8}=\frac{A_{\mathrm{vd}}/A_{\mathrm{hd}}}{A_{\mathrm{vn}}/A_{\mathrm{hn}}}$$

Here, *A_vn_* is the root mean square values of vibration signals of the vertical direction in the normal state; *A_vd_* is the root mean square values of vibration signals of the vertical direction in the abnormal states.

(9) $$P_{9}=\beta_{d}-\beta_{n}$$

Here, *β_n_* and *β_d_* are the skewness values in the normal state and the abnormal states, respectively. $\beta =\Sigma_{i=1}^{I}{\left(f_{i}-\bar{f}\right)}^{3}\cdot P(f_{i})/\delta^{3}I$, and *I* is the number of the spectrum line, *f̄* is the mean value of the analysis frequency $\bar{f}=\Sigma_{i=1}^{I}f_{i}\cdot P(f_{i})/\Sigma_{i=1}^{I}P(f_{i})$, *δ* is the standard deviation $\delta =\sqrt{\Sigma_{i=1}^{I}{\left(f_{i}-\bar{f}\right)}^{2}\cdot P(f_{i})/I}$.

## 3. Synthetic Detection Index (SDI)

Supposing that *x*_1_ and *x*_2_ are values of a symptom parameter (SP) calculated from the signals measured in state 1 and state 2, respectively, and conforming respectively to the normal distributions N(*μ*_1_,*σ*_1_) and N(*μ*_2_,*σ*_2_). Here, μ and σ are the average and the standard deviation of the SP. The larger the value of |*x*_2_ − *x*_1_| is, the higher the sensitivity of distinguishing the two states by the SP. Because *z* = *x*_2_ − *x*_1_ also conforms to the normal distribution N(*μ*_2_ − *μ*_1_,*σ*_1_ + *σ*_2_), there is the following density function about *z*:

(11) $$f(z)=\frac{1}{\sqrt{2\pi \left(\sigma_{1}^{2}+\sigma_{2}^{2}\right)}}\text{exp}\left\{\frac{{\left\{z-\left(\mu_{2}-\mu_{1}\right)\right\}}^{2}}{2\left(\sigma_{1}^{2}+\sigma_{2}^{2}\right)}\right\}$$

where, *μ*_2_ ≥ *μ*_1_ (the same conclusion can be drawn when *μ*_1_ *≥ μ*_2_). The probability can be calculated with the following formula:

(12) $$P_{0}=\int_{-\infty }^{0}f(z)d_{z}$$

where, 1 − *P*_0_ is called the “Discrimination Rate (*DR*)”. With the substitution:

(13) $$\mu =\frac{z-\left(\mu_{2}-\mu_{1}\right)}{\sqrt{\sigma_{1}^{2}+\sigma_{2}^{2}}}$$

into Formulas (11) and (12), the *P*_0_ can be obtained by:

(14) $$P_{0}=\frac{1}{\sqrt{2\pi }}\int_{-\infty }^{-\mathrm{DI}}\text{exp}\left(-\frac{\mu^{2}}{2}\right)d_{\mu }$$

where, the *DI* (Discrimination Index) is calculated by:

(15) $$\mathrm{DI}=\frac{\mu_{2}-\mu_{1}}{\sqrt{\sigma_{1}^{2}+\sigma_{2}^{2}}}\;\;\text{or}\;\;\mathrm{DI}=\frac{\bar{x_{2}}-\bar{x_{1}}}{\sqrt{\sigma_{1}^{2}+\sigma_{2}^{2}}}$$

It is obvious that the larger the value of the *DI*, the larger the value of the “Discrimination Rate (*DR* = 1 − *P*_0_)” will be, and therefore, the better the SP will be. Thus, the *DI* can be used as the index of the quality to evaluate the distinguishing sensitivity of the SP. The number of symptom parameters used for the diagnosis and fault types are *M* and *N*, respectively, and the synthetic detection index (*SDI*) is defined as follows:

(16) $$\mathrm{SDI}=\sum_{i=1}^{N-1}\sum_{j=i+1}^{N}\sum_{k=1}^{M}\frac{\left|\mu_{\mathrm{ik}}-\mu_{\mathrm{jk}}\right|}{\sqrt{\sigma_{\mathrm{ik}}^{2}+\sigma_{\mathrm{jk}}^{2}}}$$

## 4. Intelligent Condition Diagnosis Method Using Ant Colony Optimization (ACO)

In order to effectively and automatically distinguish faults for condition monitoring of rotating machinery, a new intelligent condition diagnosis method is proposed based on the RRSPs and the ACO. The problem of state identification for the condition diagnosis is converted into the clustering problem of the RRSPs calculated by vibration signals measured in different states, which will be solved by the ACO.

### 4.1. Ant Colony Optimization (ACO)

The ACO algorithm introduced by Marco Dorigo in his Ph.D. thesis is a population-based meta-heuristic that can be used to find approximate solutions to difficult optimization problems. The ACO algorithm is inspired by the behavior of ants while finding paths from the colony to food. Ants have no sight and are capable of finding the shortest route between a food source and their nest by chemical materials called pheromones that they leave when moving. A moving ant lays some pheromone on the ground, thus making a trail of this substance. While an isolated ant moves practically at random, an ant encountering a previously laid trail can detect it and decide with high probability to follow it and reinforce the trail with its own pheromone. What emerges is a form of an autocatalytic process through which the greater the number of ants that follow a particular trail makes that trail more attractive to be followed. The process is thus characterized by a positive feedback loop, during which the probability of choosing a path increases with the number of ants that previously chose the same path [21,22].

ACO is a kind of heuristic algorithm with global optimization, which combines distributed computing and positive feedback mechanisms and has the following virtues:

1. Stronger robustness: ACO can transplant other problems, especially all kinds of assembled optimized problems.
2. Greater ability to find the better result: The algorithm adopts the positive feedback principle, which quickens the evolution processing and does not become trapped in local optima.
3. Distributing parallelism calculating: ACO is an evolution algorithm based on ant colonies and has parallelism base on them. The individual ants can continue to exchange and transfer the information (pheromone), which can lead to a better result.
4. It is easy to combine ACO with other methods: The algorithm can integrate other enlightened methods to improve the performance of the algorithm.

### 4.2. ACO for Condition Diagnosis

Assume that *N* is the number of sample sets of vibration signals measured in *m* different states, the length of which is *n*, *N* = {*x_1_,x_2_*⋯*x_n_*}. Every sample signal has *t* indentified symptoms (in this paper, the symptoms are *P_1_∼P_9_*). Then, the clustering analysis is to divide *n* sample data into *m* states, such that the objective function *F* shown in Formula (17) is minimized:

(17) $$\text{min}\;\;F={\sum_{j=1}^{m}\sum_{i=1}^{n}\sum_{k=1}^{t}a_{ij}\left\|x_{ik}-c_{jk}\right\|}^{2}$$

where, *c_jk_* is the clustering center, and:

(18) $$c_{\mathrm{jk}}=\frac{\sum_{i=1}^{n}a_{\mathrm{ij}}x_{\mathrm{ik}}}{\sum_{i=1}^{n}a_{\mathrm{ij}}}\;\;\;\;\;\;(j=1,2\cdots m;k=1,2\cdots t)$$

(19) $$a_{\mathrm{ij}}=\{\begin{array}{cc} 1 & \mathrm{if}\;x_{i}\;\in \mathrm{state}\;j\\ 0 & \mathrm{if}\;x_{i}\notin \mathrm{state}\;j \end{array}\;\;\;\;\;\;\;\;\;\;\;(i=1,2\cdots n;j=1,2\cdots m)$$

**Figure 2.**
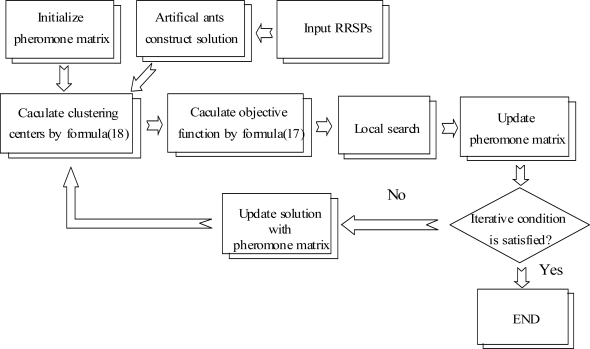
Flowchart of ACO for condition diagnosis.

In this paper, the procedure for applying the ACO for the condition diagnosis is proposed as shown in Figure 2, and the procedure is explained as follows:

1. RRSPs used for reflecting the features of sample signals are inputted into the ACO.
2. Sample signals are randomly classified by artificial ants (artificial ants construct solutions), and the pheromone matrix is initialized.
3. According to the solutions, clustering centers are calculated by Formula (18), and the object function of every solution is calculated by Formula (17).
4. Local search (refer to Section 4.4).
5. The pheromone matrix is updated (refer to Section 4.5).
6. According to pheromone matrix, artificial ants update the solutions (refer to Section 4.3).
7. Steps (3–6) are looped until the ending condition is satisfied.

### 4.3. Construction and Update of Solutions

In the ACO, every artificial ant will construct the solution *S* with a length of *n* and *S* = {*c_i_*|*I* = 1,2⋯*n*}, *c_i_* = 1,2⋯*m*, where *c_i_* is the classification result of sample *x_i_*. That is, if *c_i_* = *j*, then *x_i_* is the output vibration data in state *j*. At the start of the ACO, the solutions *S* are randomly constructed by artificial ants, and with the increase of the iteration number, artificial ants update the solutions incessantly according to the pheromone matrix information, followed by the principles given as follows:

(20) $$S=\text{argmax}\left\{\tau_{\mathrm{ij}}\times {\left[\eta_{\mathrm{ij}}\right]}^{\beta }\right\}\;\mathrm{if}\;q\leq q_{o}$$

(21) $$\eta_{\mathrm{ij}}=\frac{1}{d_{\mathrm{ij}}}$$

where, *d_ij_* is the Euclidean distance between clustering center *j* and sample *x_i_*, and:

(22) $$d_{\mathrm{ij}}=\sqrt{\sum_{k=1}^{t}{\left(x_{\mathrm{ik}}-c_{\mathrm{jk}}\right)}^{2}}$$

Here, *q* is a value chosen randomly with a uniform probability between 0∼1, *q_o_* is constant, 0 < *q_o_* < 1, *τ_ij_* represents the pheromone concentration of sample. *x_i_* associated with the state *j* and *β* is a parameter that determines the relative importance of heuristic information (the choice of *β* is determined experimentally, and *β* > 0).

If *q_o_* < *q*, the artificial ants choose the state for sample *x_i_* by the conversion probability *p_ij_* given as follows:

(23) $$p_{\mathrm{ij}}=\frac{\tau_{\mathrm{ij}}\times {\left[\eta_{\mathrm{ij}}\right]}^{\beta }}{\sum_{s=1}^{m}\tau_{\mathrm{is}}\times {\left[\eta_{\mathrm{is}}\right]}^{\beta }}$$

### 4.4. Local Search

To improve the efficiency and accelerate the convergence speed of the ACO, the method of local search for the ACO is presented. The local search method is conducted on all solutions or some solutions [23,24]. In this paper, the latter is applied, that is, local search is implemented only for the ten solutions with smaller objective functions. The execution process of the local search for the ACO is as follows:

1. All solutions are arranged in ascending order according to the values of the objective function.
2. Random data *W_i_* {*i* = 1,2⋯*n*} for every sample are produced automatically.
3. A weight *P* is set, and 0 < *P* < 1.
4. *P* is compared with *W_i_*, if *P* > *W_i_*, and then the sample *x_i_* is reclassified.
5. The Euclidean distance between sample *x_i_* and every clustering center is calculated, and the shortest distance is for the class of sample *x_i_*.
6. Formula (17) is used to compute the objective function again and compare it with the former objective function values. If the new one is lower than the former one, the new solution sets are kept, or the former solution sets are reduced.
7. Steps (2–6) are looped until the ten solutions are calculated.

### 4.5. Update Pheromone Matrix

Dorigo proposed three different models: the ant-cycle system, the ant-quantity system and the ant-density system [25]. In this research, the ant-cycle system is used to update the pheromone. In the ant-cycle system, the pheromone is released after the artificial ant builds all information. It utilizes all information. However, the other two systems utilize only partial information. Thus, this system is better than the ant-quantity system and the ant-density system. The pheromone will be updated by the ten artificial ants that have smaller object functions, and the updating principle is as follows:

(24) $$\tau_{\mathrm{ij}}(l+1)=(1-\rho )\tau_{\mathrm{ij}}(l)+\sum_{a=1}^{10}\Delta \tau_{\mathrm{ij}(a)}$$

(25) $$\Delta \tau_{\mathrm{ij}(a)}=\{\begin{array}{cc} \frac{1}{F_{a}} & x_{i}\in \mathrm{state j}\\ 0 & \mathrm{otherwire} \end{array}$$

(26) $$F_{a}={\sum_{j=1}^{m}\sum_{i=1}^{n}\sum_{k=1}^{t}\left\|x_{ik}-c_{jk}\right\|}^{2}$$

Here, *τ_ij_* represents the pheromone concentration of sample. *x_i_* associated with the state *j*, *ρ* is the decay parameter of the pheromone and, to prevent pheromone excessive accumulation 0 < *ρ* < 1, Δ*τ_ij(a)_* is the pheromone values of artificial ant *a*.

From Formulas (24–26), if sample *x_i_* ∈ state *j*, with increasing iteration number, then the pheromone *τ_ij_* becomes greater and finally approaches the saturation level. On the contrary, if sample *x_i_* ∉ state *j*, with increasing iteration number, then the pheromone *τ_ij_* becomes smaller and finally approaches 0.

## 5. Diagnosis and Application

In this section, the application of condition diagnosis to a centrifugal fan is shown to verify that the method proposed in this paper is effective. To illustrate the effectiveness of the proposed method in the diagnosis of structural faults of rotating machinery, we also compare it with the conventional NN method.

### 5.1. Experimental System

**Figure 3.**
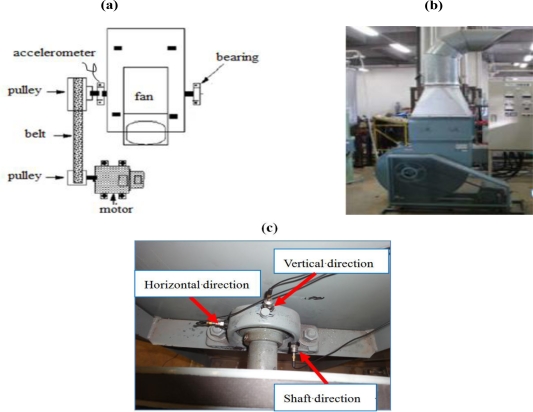
Experiment system for the fault diagnosis **(a)** Illustrate of the centrifugal fan, **(b)** The centrifugal fan in the field, **(c)** Measurement points of the accelerometer.

**Figure 4.**
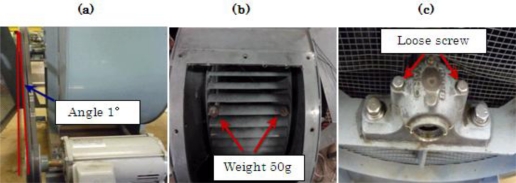
Structural faults **(a)** Misalignment state, **(b)** Unbalance state, **(c)** Looseness state.

**Figure 5.**
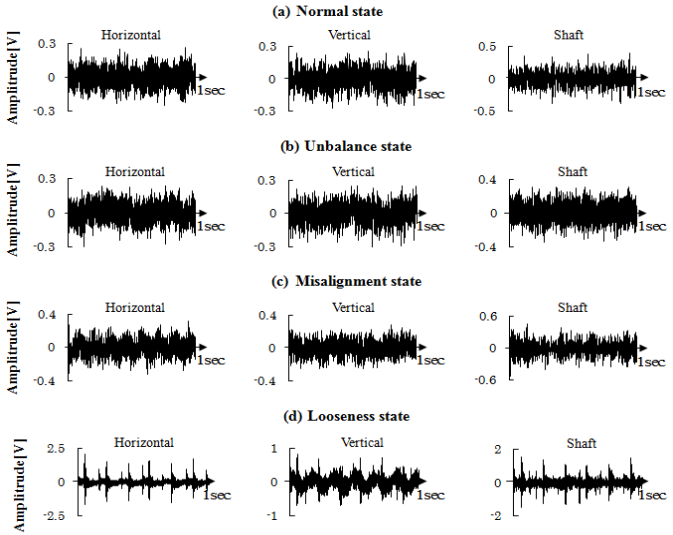
Raw vibration signals in time domain **(a)** Normal state, **(b)** Unbalance state, **(c)** Misalignment state, **(d)** Looseness state.

**Figure 6.**
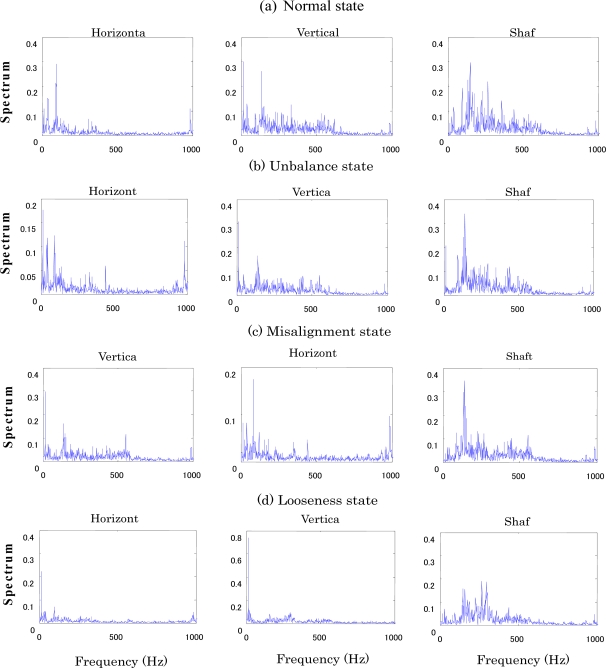
Raw vibration signals in frequency domain. **(a)** Normal state, **(b)** Unbalance state, **(c)** Misalignment state, **(d)** Looseness state.

The centrifugal fan for the diagnosis test and structural faults such as the normal (N), unbalance (UN), misalignment (M) and looseness (L) states is shown in Figures 3 and 4, respectively. The three accelerometers (PCB MA352A60) with a bandwidth from 5 Hz to 60 kHz and 10 mV/g output were used to measure the vibration signals of the horizontal, vertical and shaft directions in the normal (N), unbalance (UN), misalignment (M) and looseness (L) states, respectively. The vibration signals measured by the accelerometers were transformed into the signal recorder (Scope Coder DL750) after being magnified by the sensor signal conditioner (PCB ICP Model 480C02). The original vibration signals in time domain and frequency domain are shown in Figure 5 and Figure 6 respectively. These signals were measured at a constant speed (600 rpm). The sampling frequency of the signal measurement was 50 kHz, and the sampling time was 20 s.

The RRSPs calculated by Formulas (1–9), which have high sensitivity for the condition diagnosis, are selected by *SDI*, as shown in Formula (16). Table 1 lists the *SDI*s of the RRSPs. The maximum value (94.6) of *SDI* was obtained in the case of the combination of *P*_6_, *P*_7_ and *P*_8_, and, when *P*_6_, *P*_7_ and *P*_8_ are singly used for distinguishing each state, the *DI*s were larger than 1.75. All of the discrimination rate of *P*_6_, *P*_7_ and *P*_8_ were larger than 95%. The combination of *P*_6_, *P*_7_ and *P*_8_ has high sensitivity for the structural faults diagnosis of the centrifugal fan. Table 2 shows the *DI*s of *P*_6_, *P*_7_ and *P*_8_.

### 5.2. Diagnosis by the Proposed Method

The main procedure for fault diagnosis using RRSPs and ACO was introduced in Section 1 (refer to Figure 1). First, the vibration signals are measured in each known state. Second, the RRSPs are calculated using Formulas (1–9). The highly sensitive RRSPs (*P*_6_, *P*_7_, *P*_8_) are selected for condition diagnosis by the *SDI*. Third, the ACO is trained with *P*_6_, *P*_7_, *P*_8_, and the optimal clustering centers are obtained. Lastly, the condition of the centrifugal fan can be diagnosed by the trained ACO and RRSPs.

When a rotating machine is in a looseness state, the spectrum values in the high frequency region are obviously higher than in the misalignment and unbalance states. The symptom parameter *P*_6_ indicates the ratio of the spectrum values between high frequency domain and low frequency domain. *P*_6_ has high sensitivity for distinguishing the looseness state from other states. When a rotating machine is in a misalignment state, the vibration level in the shaft direction is stronger than in looseness and unbalance states. The symptom parameter *P*_7_ is the ratio of the vibration level between the shaft direction and the horizontal direction, so *P*_7_ has high sensitivity for distinguishing the misalignment state from other states. When a rotating machine is in an unbalance state, the vibration level of the vertical direction is stronger than in looseness and misalignment states. The symptom parameter *P*_8_ is the ratio of the vibration level between the horizontal direction and the horizontal direction, so *P*_8_ has high sensitivity for distinguishing the unbalance state from other states. Therefore, the combination of *P*_6_, *P*_7_ and *P*_8_ has high sensitivity for the structural faults diagnosis of the centrifugal fan.

**Figure 7.**
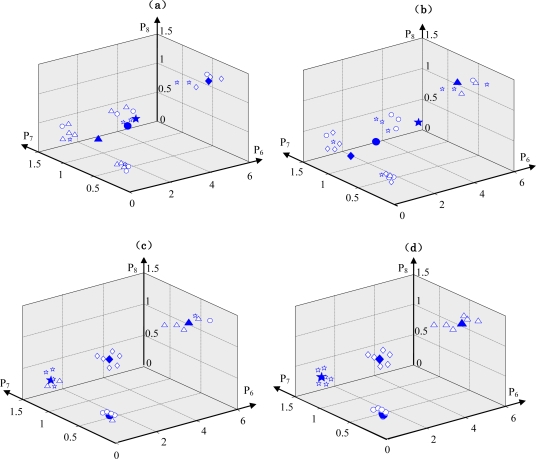
The change of the clustering centers while training the ACO for the condition diagnosis of the centrifugal fan. **(a)** At the start of the ACO, **(b)** after 50 iterations, **(c)** after 100 iterations and **(d)** after 150 iterations.

In this research, the state identification for the condition diagnosis is converted to a clustering problem for the values of the RRSPs calculated from vibration signals measured in different states of the centrifugal fan. The ACO automatically finds the optimal clustering centers and classify all sample data according to the amount of information around the clustering centers. The purpose of training the ACO is the acquisition of optimum clustering centers. *P*_6_, *P*_7_ and *P*_8_ calculated using the vibration signals measured in each known state were input into the ACO. After about 150 iterations, the ACO converged to the optimum clustering centers. Table 3 lists the parameters values for training the ACO, and Figure 7 shows the change of the clustering centers while training the ACO for the condition diagnosis of the centrifugal fan.

Here, the symbols ⋄, ○, ☆ and Δ express the value samples of RRSPs in the normal state, unbalance state, misalignment state and looseness state, respectively, and the big symbols represent their clustering centers.

In the training process of the ACO, at first, the sample data are classified into normal, unbalance, misalignment and looseness states randomly. The clustering centers and the sum of the spatial distance between every sample data and the clustering centers are calculated by Formulas (17–19). With increasing iterations, the pheromones are updated incessantly, and according to the pheromone information, the classification of the sample data and clustering centers are also updated by artificial ants. Finally, the optimal clustering centers with a minimum sum of spatial distances are calculated. As an example, parts of the training data and their clustering centers are shown in Table 4.

**Figure 8.**
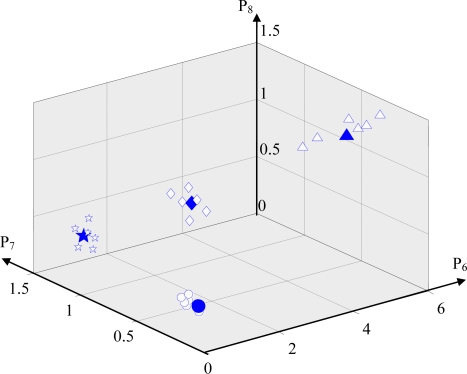
**C**lassification of the test signals.

**Figure 9.**
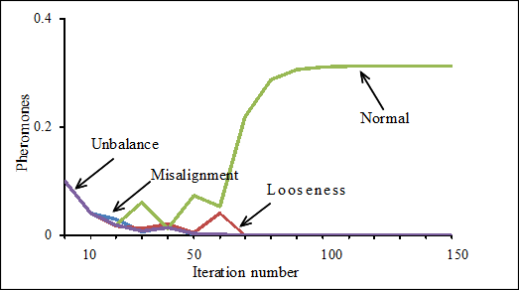
Pheromones for distinguishing the normal state from abnormal states.

Here, x is the coordinate value of clustering center on the *P*_6_ axis, y is the coordinate value of clustering center on the *P*_7_ axis, z is the coordinate value of clustering center on the *P*_8_ axis. After training the ACO, to verify the diagnostic capability of the proposed method in this paper, the test data measured in each known state that had not been used to train the ACO were used. When inputting the test data into the trained ACO, the ACO classified the test data according to the information of the optimum clustering centers shown in Table 4 and correctly and quickly output identification results based on the pheromone values of the corresponding states. As an example, Figure 8 shows the parts of the test data classified according to the information of the optimum clustering centers shown in Table 4. Figure 9 shows the change of the pheromones for distinguishing the normal state from abnormal states with increasing iterations. Figure 9 shows that the pheromone of the normal state gradually increases and finally approaches the saturation level. On the contrary, the pheromones of each abnormal state gradually decrease and finally approach 0. Some diagnosis results are listed in Table 5. These results verified the efficiency of the intelligent diagnosis method using RRSPs and the ACO proposed in this paper.

**Figure 10.**
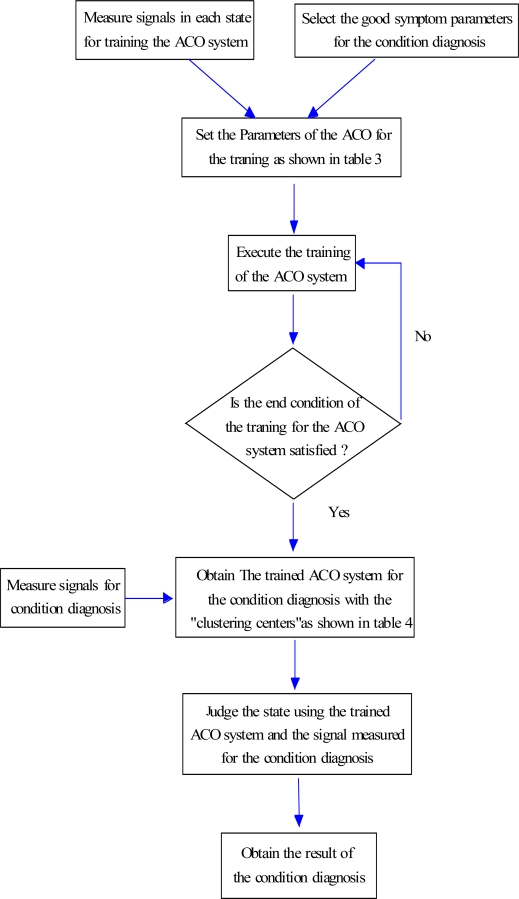
The flowchart of the condition diagnosis using the ACO system.

To summarize the condition diagnosis method proposed in this paper for a rotating machine, Figure 10 shows the flowchart of the method. The state of the rotating machine can be quickly and automatically diagnosed by using the RRSPs and the ACO system, as shown in Figure 10.

### 5.3. Diagnosis by Neural Network

**Figure 11.**
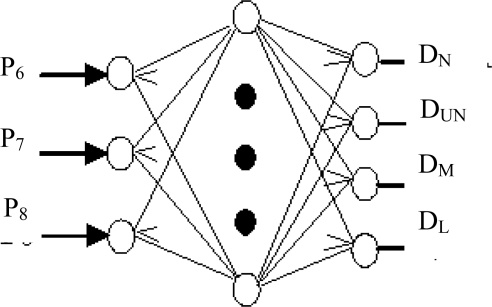
NN for pattern recognition in fault diagnosis.

**Figure 12.**
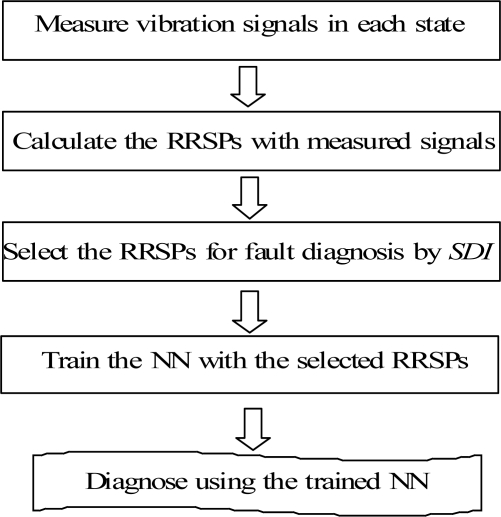
Flowchart of fault diagnosis by the NN.

In order to compare the performances of the ACO and a neural network (NN) for the condition diagnosis, a NN was also built, which consisted of the input layer, the hidden layer and the output layer, as shown in Figure 11. The parameters entered into the input layer of the NN were RRSPs. The number of neurons in the hidden layer was eighty, and the outputs in the last layer were D_N_, D_UN_, D_M_, D_L_, which indicate the normal (N), unbalance (UN), misalignment (M) and looseness (L) states, respectively. The flowchart of fault diagnosis by the NN is shown in Figure 12.

In this paper, when the NN was applied to fault diagnosis, the diagnostic knowledge (teaching data) for the NN was acquired by probability theory using the probability distributions of the RRSPs (*P*_6_, *P*_7_, *P*_8_) calculated by the vibration signals measured in each known state and selected by *SDI*. An example for obtaining the possibility grade *D_N_* used to judge the normal state is shown as follows. *p_iN_* indicate the value RRSP of the normal state (N), and its mean value and standard deviation are *p̄_iN_* and *S_iN_*, respectively. *D_iN_* is the possibility grades of the normal state (0 or 1). The training data for distinguishing the normal state from another state are calculated as follows:

(27) $${\bar{p}}_{\mathrm{iN}}-{2S}_{\mathrm{iN}}\leq p_{\mathrm{iN}}\leq {\bar{p}}_{\mathrm{iN}}+{2S}_{\mathrm{iN}}\to D_{\mathrm{iN}}=1$$

(28) $$\begin{array}{ccc} p_{\mathrm{iN}}<{\bar{p}}_{\mathrm{iN}}-2S_{\mathrm{iN}} & \mathrm{or} & {\bar{p}}_{\mathrm{iN}}+{2S}_{\mathrm{iN}}<p_{\mathrm{iN}}\to D_{\mathrm{iN}}=0 \end{array}$$

For condition diagnosis using two or more RRSPs, the possibility grades *D_N_* are defined as follows, and *M* is the number of RRSPs:

(29) $$D_{N}=\underset{i=1∼M}{\mathrm{Min}}\left\{D_{\mathrm{iN}}\right\}$$

To train the NN, the training data obtained by the method mentioned above were input into the NN. After about 10,000 iterations, the NN converged. As an example, part of the acquired training data for the NN is shown in Table 6.

After training the NN, the faults of the centrifugal fan were diagnosed with the learned NN. To compare the efficiency of the method proposed in this research with the NN, the same test data used in the ACO were input into the learned NN. As an example, some of the diagnosis results are shown in Table 7. The symbol × indicate the case in which the NN cannot identify the fault type.

According to the diagnosis results shown in Table 7, the unbalance and looseness states of the centrifugal fan cannot be correctly identified by the NN because the vibration signals contain strong noise and there exist ambiguous relationships between the RRSPs and the fault types.

The reasons of the low diagnosis accuracy by using the NN are thought to be: (1) Conventional NN cannot reflect the possibility grades of the ambiguous diagnosis problems. (2) Conventional NN will never converge when the symptom parameters inputted in the 1st layer have the same values in different states.

## 6. Conclusions

In order to detect faults and distinguish fault types at an early stage, this paper proposes a new method for diagnosing structural faults of rotating machinery developed by using relative ratio symptom parameters (RRSPs) and ant colony optimization (ACO). The main conclusions can be summarized as follows:

1. The nine symptom parameters called “relative ratio symptom parameters” in the low-frequency domain were defined for reflecting the features of vibration signals measured in each state.
2. The state identification for the condition diagnosis of rotating machinery was converted to a clustering problem of the values of the relative ratio symptom parameters (RRSPs) in the low-frequency domain, calculated from vibration signals in different states of the machine. Ant colony optimization (ACO) was also introduced for this purpose.
3. The synthetic detection index (*SDI*) on the basis of statistical theory was also defined to evaluate the applicability of the RRSPs. The *SDI* can be used to select better RRSPs for the ACO.
4. A comparison was made between the proposed method and a neural network (NN), and the practical example of faults diagnosis of the centrifugal fan verified the effectiveness of the proposed method. The diagnosis results showed that the structural faults which occur in the centrifugal fan, such as unbalance, misalignment and looseness states, *etc.*, were automatically and effectively identified by the proposed method. However, these faults could not be correctly identified by the NN.

In this paper, we have verified the efficiency of the ACO diagnosis system in order to detect faults and distinguish fault types at an early stage. For the future study, we will apply the method to detect and diagnose faults at every fault stages, such as initial stage fault, moderate stage fault and serious fault *etc.*

## Figures and Tables

**Table 1.** *SDI*s of each RRSP.

**RRSPs**	***SDI***	***DI_max_***	***DI_min_***
P_1_P_2_P_3_	48.67	9.8	0.31
P_1_P_2_P_4_	27.26	5.67	0.41
…	…	…	…
P_6_P_7_P_8_	94.60	9.66	1.75
P_6_P_7_P_9_	81.16	9.66	0.27
P_6_P_8_P_9_	69.04	7.83	0.27
P_7_P_8_P_9_	65.24	9.66	0.27

**Table 2.** *DI*s of *P*_6_, *P*_7_ and *P*_8_.

***DI***	**P_6_**	**P_7_**	**P_8_**
*DI_N-M_*	7.65	5.80	3.36
*DI_N-L_*	6.63	6.18	2.40
*DI_N-UN_*	5.42	2.71	5.74
*DI_M-L_*	7.83	1.75(*DI_min_*)	1.85
*DI_M-UN_*	2.66	9.66(*DI_max_*)	2.13
*DI_L-UN_*	7.57	8.87	6.37

**Table 3.** Parameters of the ACO.

**Contents**	**Values**
Weight value for updating solution *q*_o_	0.5
Parameter of heuristic information *β*	0.6
Weight value of local search *P*	0.2
Decay parameter of pheromone *ρ*	0.1

**Table 4.** Parts of acquired data of diagnosis for the ACO. **(a)** Normal state, **(b)** Unbalance state, **(c)** Misalignment state, **(d)** Looseness state.

**(a)** Normal state					
RRSPs			Clustering Center		
P_6_	P_7_	P_8_	x	y	z
1.126	1.033	0.749	1.056	1.005	0.913
0.941	0.938	1.099			
1.260	0.967	0.967			
…	…	…			

**(b)** Unbalance state					
RRSPs			Clustering Center		
P_6_	P_7_	P_8_	x	y	z
0.430	0.772	0.181	0.501	0.827	0.223
0.525	0.779	0.244			
0.588	0.851	0.181			
…	…	…			

**(c)** Misalignment state					
RRSPs			Clustering Center		
P_6_	P_7_	P_8_	x	y	z
0.352	1.366	0.319	0.342	1.397	0.416
0.329	1.307	0.452			
0.302	1.440	0.506			
…	…	…			

**(d)** Looseness state					
RRSPs			Clustering Center		
P_6_	P_7_	P_8_	x	y	z
5.748	1.461	0.595	5.256	1.533	0.581
4.799	1.636	0.534			
4.963	1.481	0.540			
…	…	…			

**Table 5.** Diagnosis result using proposed method.

**RRSPs**			**Pheromones**				**Judge**

**P_6_**	**P_7_**	**P_8_**	**N**	**UN**	**M**	**L**	

1.084	0.937	0.854	0.296	0	0	0	N
0.976	1.114	0.901	0.296	0	0	0	N
0.430	0.772	0.181	0	0.296	0	0	UN
0.509	0.856	0.255	0	0.296	0	0	UN
0.345	1.445	0.277	0	0	0.296	0	M
0.388	1.399	0.563	0	0	0.296	0	M
5.049	1.589	0.527	0	0	0	0.296	L
5.530	1.514	0.618	0	0	0	0.296	L
…	…	…	…	…	…	…	…

**Table 6.** Training data for the NN.

**RRSPs**			**States**			

**P_6_**	**P_7_**	**P_8_**	**N**	**UN**	**M**	**L**

0.871	0.913	0.662	1	0	0	0
1.051	1.014	0.884	1	0	0	0
1.231	1.115	1.106	1	0	0	0
0.290	1.311	0.241	0	1	0	0
0.337	1.398	0.412	0	1	0	0
0.384	1.485	0.583	0	1	0	0
3.994	1.403	0.498	0	0	1	0
5.247	1.541	0.593	0	0	1	0
6.500	1.679	0.688	0	0	1	0
0.385	0.768	0.145	0	0	0	1
4.488	0.843	0.215	0	0	0	1
0.591	0.918	0.285	0	0	0	1
…	…	…	…	…	…	…

**Table 7.** Diagnosis result using the NN.

**RRSPs**			**Fault Types**				**Judge**

**P_6_**	**P_7_**	**P_8_**	**N**	**UN**	**M**	**L**	

1.084	0.937	0.854	0.988	0	0.018	0.009	N
0.976	1.114	0.901	0.996	0	0.004	0.013	N
0.430	0.772	0.181	0.001	0.560	0	0.444	×
0.509	0.856	0.255	0.001	0.562	0	0.443	×
0.345	1.445	0.277	0.005	0	0.993	0.005	M
0.388	1.399	0.563	0.005	0	0.992	0.007	M
5.049	1.589	0.527	0.001	0.561	0	0.443	×
5.530	1.514	0.618	0.001	0.517	0	0.418	×
…	…	…	…	…	…	…	…
